# Rutaecarpine Inhibits Doxorubicin-Induced Oxidative Stress and Apoptosis by Activating AKT Signaling Pathway

**DOI:** 10.3389/fcvm.2021.809689

**Published:** 2022-01-05

**Authors:** Zi-Qi Liao, Yi-Nong Jiang, Zhuo-Lin Su, Hai-Lian Bi, Jia-Tian Li, Cheng-Lin Li, Xiao-Lei Yang, Ying Zhang, Xin Xie

**Affiliations:** ^1^Department of Cardiology, First Affiliated Hospital of Dalian Medical University, Dalian, China; ^2^Institute of Cardiovascular Diseases, First Affiliated Hospital of Dalian Medical University, Dalian, China

**Keywords:** rutaecarpine, doxorubicin, oxidative stress, apoptosis, cardiotoxicity, AKT

## Abstract

Patients with cancer who receive doxorubicin (DOX) treatment can experience cardiac dysfunction, which can finally develop into heart failure. Oxidative stress is considered the most important mechanism for DOX-mediated cardiotoxicity. Rutaecarpine (Rut), a quinazolinocarboline alkaloid extracted from *Evodia rutaecarpa* was shown to have a protective effect on cardiac disease. The purpose of this study is to investigate the role of Rut in DOX-induced cardiotoxicity and explore the underlying mechanism. Intravenous injection of DOX (5 mg/kg, once a week) in mice for 4 weeks was used to establish the cardiotoxic model. Echocardiography and pathological staining analysis were used to detect the changes in structure and function in the heart. Western blot and real-time PCR analysis were used to detect the molecular changes. In this study, we found that DOX time-dependently decreased cardiac function with few systemic side effects. Rut inhibited DOX-induced cardiac fibrosis, reduction in heart size, and decrease in heart function. DOX-induced reduction in superoxide dismutase (SOD) and glutathione (GSH), enhancement of malondialdehyde (MDA) was inhibited by Rut administration. Meanwhile, Rut inhibited DOX-induced apoptosis in the heart. Importantly, we further found that Rut activated AKT or nuclear factor erythroid 2-related factor 2 (Nrf-2) which further upregulated the antioxidant enzymes such as heme oxygenase-1 (HO-1) and GSH cysteine ligase modulatory subunit (GCLM) expression. AKT inhibitor (AKTi) partially inhibited Nrf-2, HO-1, and GCLM expression and abolished the protective role of Rut in DOX-induced cardiotoxicity. In conclusion, this study identified Rut as a potential therapeutic agent for treating DOX-induced cardiotoxicity by activating AKT.

## Introduction

Doxorubicin (DOX) is an effective chemotherapeutic drug that is widely used in treating several solid tumors and malignant hematologic diseases (1). However, DOX-induced cardiotoxicity is an insurmountable barrier that can limit its clinical application. The molecular mechanism of DOX-induced cardiotoxicity has been extensively investigated, and now oxidative stress, autophagy, mitochondrial dysfunction, and apoptosis pathways were commonly recognized as the underlying mechanism of DOX-induced cardiotoxicity (2). Previous studies have proved that inhibition of oxidative stress dramatically alleviated DOX-induced cardiotoxicity in the heart (3). Therefore, exploring novel drugs to inhibit DOX-induced oxidative stress thereby improving patients' quality of life is urgently needed.

AKT is an essential signaling pathway regulating cell proliferation, glucose metabolism, oxidative stress, and also autophagy (4). Recent studies reported DOX inhibited PI3K/AKT signaling pathway in mice heart, and inhibition of PI3K/AKT aggravated DOX-induced cell death and heart failure (5), whereas pharmacological activation of PI3K/AKT attenuated DOX-induced cardiotoxicity (6). Nrf-2 is an essential antioxidative gene that inhibits oxidative stress *via* upregulation of the intracellular antioxidant enzymes such as HO-1 and GCLM, which promote glutathione (GSH) generation (7). Increased GSH would scavenge the excess reactive oxygen species (ROS) in DOX-treated cardiomyocytes and inhibit various forms of cell death including apoptosis and necroptosis (8, 9). The activation of PI3K/AKT is noteworthy as it was able to increase Nrf-2 and GCLM expression in the heart of DOX-treated mice (10, 11). Therefore, targeting the PI3K/AKT signaling pathway to prevent oxidative stress could be a prudent strategy in Dox-induced cardiotoxicity. Rutaecarpine (8,13-dihydroindolo-(2′,3′:3,4)pyrido(2,1-b)quinazolin-5(7H)-one) is a quinazolinocarboline alkaloid that is extracted from the traditional Chinese herb *Evodia rutaecarpa* (also named Wu Zhu Yu). It exerts beneficial roles in treating several diseases including hypertension (12), cardiac hypertrophy (13), cardiac ischemia-reperfusion injury (14), diabetes (15), and tumor (16). The cardioprotective effects of Rut were mainly attributed to its vasodilatory, antiplatelet activation, antioxidant effect, and anti-inflammatory effects (17). The antioxidant effect of Rut was to a large extent due to its regulatory role in antioxidant enzymes and NADPH oxidase. It has been reported that Rut protected hepatotoxicity by upregulating antioxidant enzymes through the Nrf-2/ARE pathway (18) and alleviated hypoxia-reoxygenation induced cardiomyocytes apoptosis through inhibiting NADPH oxidase expression (19). Since Rut has been shown to activate PI3K/AKT signaling pathway (15), and PI3K/AKT was the upstream signal of Nrf-2 (20), in this study, we investigated whether Rut protected against DOX-induced cardiotoxicity.

In this study, we showed that rutaecarpine alleviated DOX-induced cardiac dysfunction and cardiomyocyte death by inhibition of oxidative stress and the antioxidant effect of Rut probably due to the activation of the AKT/Nrf-2 signaling pathway. Thus, we concluded that Rut may become a novel potential drug to mitigate DOX-induced cardiotoxicity.

## Materials and Methods

### Antibodies and Reagents

Antibodies for total AKT and phosphorylated AKT were purchased from Cell Signaling Technology (Danvers, MA, USA). Antibodies for GCLM, Nrf-2, HO-1, cleaved caspase-3, Bax, Bcl-2, and GAPDH were purchased from Proteintech (Wuhan, Hubei, China). DOX was obtained from Absin (Beijing, China), and the rutaecarpine was obtained from Chengdu Must Bio-technology (Chengdu, Sichuan, China). The AKT inhibitor (AKTi), wheat germ agglutinin (WGA), and dihydroethidium (DHE) were purchased from Sigma-Aldrich (Santa Clara, CA, USA). Hematoxylin-eosin/HE and Masson's trichrome staining kit were from Solarbio (Beijing, China). The malondialdehyde (MDA) assay kit and total SOD assay kit were purchased from Nanjing Jiancheng Bioengineering Institute (Nanjing, Jiangsu, China). The GSH assay kit was purchased from Beyotime Biotechnology (Shanghai, China). TRIzol was obtained from Invitrogen (Carlsbad, CA, USA). All primers used in our laboratory were purchased from Sangon Biotech (Shanghai, China).

### Animals and Treatments

Male C57BL/6 mice aged 8–10 weeks old were purchased from Vital River Laboratory Animal Technology (Beijing, China) and were kept in pathogen-free and individual ventilated cages. All mice were grown in the standard humidity or temperature-controlled environment (70% relative humidity, 22°C) and a 12:12-h light-dark cycle. Mice were divided into six groups and subjected to the following protocols: (1) In the control group, mice were treated with saline containing 0.5% dimethyl sulfoxide(DMSO); (2) In the Rut group, Rut was dissolved in saline contained 0.5% Tween 80, and then mice received intragastric administration of 40 mg/kg Rut every day for 4 weeks; (3) In DOX group, mice were intravenously treated with DOX (5 mg/kg, dissolved in saline contained 0.5% DMSO) once per week for 2 or 4 weeks; (4) In DOX plus Rut (low dose) group, mice treated with DOX and then at the same day received intragastric administration of 20 mg/kg Rut every day for 4 weeks; (5) In DOX plus Rut (high dose) group, mice were treated with DOX and parallelly received intragastric administration of 40 mg/kg Rut every day for 4 weeks; (6) In DOX plus Rut and AKTi group, mice were intraperitoneally injected with AKTi (20 mg/kg) every other day for 4 weeks from the first day before DOX and Rut administration. Because the long-term use of DOX can result in severe side effects, we measured the changes in the food uptake, body weight, and heart function every 2 weeks. Finally, all the mice were sacrificed. The heart was harvested and then analyzed by histopathology, western blot, and real-time PCR.

### Echocardiographic Assessment

After drug treatment for 2 and 4 weeks, mice were subjected to about 2% isoflurane mixed with 100% O^2^ by inhalation to induce anesthesia as we described previously (21). Trans-thoracic two-dimensional M mode echocardiography at different time points was determined using a 30-MHz probe (Vevo 770 imaging system, VisualSonics, Toronto, Canada). The heart rate was maintained at 450–500 beats per min, and then the heart rate, left ventricular ejection fraction (LVEF), LV fractional shortening (LVFS), LV posterior wall (LVPW), and LV anterior wall (LVAW) at diastole and systole were measured.

### Real-Time PCR

Total RNA of mice hearts was extracted using TRIzol Reagent (Invitrogen, Carlsbad, CA) according to the manufacturer's recommendations. Double-stranded cDNA synthesis was performed using a TaKaRa cDNA synthesis kit (TaKaRa). Real-time PCR analysis was performed using the SYBR Select Master Mix according to the manufacturer's instructions (Applied Biosystems, Thermo Fischer Scientific). The mRNA of target gene expressions was normalized to GAPDH, and the relative value to sample in the control group is given by 2^−Δ*ΔCT*^. The primer pairs were as follows: ANF, 5′-TACAGTGCGGTG TCCAACACAG-3′ and 5′-TGCTTCCTCAGTCTGCTCACTC-3′; BNP, 5′-TCCTAGCCA GTCTCCAGAGCAA-3′ and 5′-GGTCCTTCAAGAGCTGTCTCTG-3′; collagen I, 5′-GAGTACTGGATCG ACCCTAACCA-3′ and 5′-GACGGCTGAGTAGGGAACACA-3′; collagen III, 5′-TCCCCTGGAATCTGTGAATC-3′ and 5′-TGAGTCGAATTGGGGAGAAT-3′; GAPDH, 5′-AGGTCGGTGTGAACGGATTTG-3′ and 5′-TGTAGACCATGTAGTTGAGGTCA-3′.

### Western Blotting

The cardiac tissue was cracked with ice-cold RIPA buffer containing a 1% protease inhibitor cocktail. Equal amounts (20–30 μg) of protein were subjected to SDS-PAGE gel for electrophoresis and transferred onto PVDF membrane by Bio-Rad western blotting system. The membranes were blocked with 5% dry milk (dissolved in PBS buffer containing 0.1% Tween 20) for 60 min at room temperature and then incubated with anti-GCLM, HO-1, p-AKT, AKT, Nrf-2, cleaved caspase-3, Bax, Bcl-2, and GAPDH overnight at 4°C. After washing by TBST buffer for 3 times, the membrane was incubated with the horseradish peroxidase-conjugated secondary antibodies for 1 h at room temperature. Finally, the immunoreactive bands were detected by Gel-Pro 4.5 Analyzer (Media Cybernetics), and the protein expression level was quantified using the ImageJ software.

### Histological Analysis

The fresh hearts were fixed in a 4% paraformaldehyde solution for 24 h. After dehydration in xylene and ethanol, the heart samples were embedded in paraffin, cut into 5 μM thickness, and then subjected to hematoxylin and eosin (H&E) and Masson's trichrome staining assay kit according to the manufacturer's instruction (Solarbio, Beijing). To assess the size of cardiomyocytes, the heart sections were stained with 50 μg/ml TRITC-labeled WGA in PBS buffer at 37°C for 1 h and then subjected to three times washing. After antifluorescence quenching agent treatment, the digital images were photographed using a fluorescence microscope (BX51, OLYMPUS, Japan), and the cardiomyocyte's surface area was analyzed by Image-Pro Plus software.

### TUNEL Staining

The slides obtained were processed for a TUNEL assay to detect fragmented nuclei in the myocardium. The annexin V-EGFP apoptosis detection kit was used to detect apoptotic cells according to the manufacturer's protocol. Briefly, the paraffin-sectioned tissues were dewaxed and rehydrated, and then the slides were pretreated with 3% H_2_O_2_ and incubated with the reaction mixture containing TdT enzyme and biotin-11-dUTP at 37°C for 1 h. One hundred microliter of horseradish peroxidase-conjugated streptavidin (HRP-labeled avidin working fluid) was added to each slide and incubated at 37°C for 30 min. Reaction products were visualized with diaminobenzidine (DAB) plus substrate–chromogen solution for an appropriate time. The sections stained were visualized using a fluorescence microscope (BX51, OLYMPUS, Japan). The apoptotic cell number in each section was calculated by counting the number of TUNEL-positive apoptotic cells in five random fields.

### ROS Detection

To evaluate ROS generation in the heart, the heart sections were stained with 10 μM DHE at 37°C for 30 min. The superoxide anion (${\text{O}}_{2}^{-}$) in the heart was able to stain red fluorescence. After the heart sections were washed three times with PBS buffer, the digital images were photographed, and the fluorescence intensity of DHE was quantified by Image-Pro Plus software.

### Determination of SOD and MDA Activity

The myocardial tissue homogenate was broken by ultrasound following 2,000 g centrifugation for 5 min at 4°C, and the supernatant was prepared for the next examination. The levels of superoxide dismutase (SOD) and MDA of each group were analyzed using an automated microplate reader according to the instruction of their corresponding assay kits (Nanjing Jiancheng Bioengineering Institute, China).

### GSH Content Assay

The GSH assay kit (Beyotime Biotech) was used to detect the GSH contents in the heart after drug treatments. In brief, cardiac tissue (100 μg) was lysed in the protein removal solution S provided by the kit and centrifuged at 12,000 g at 4°C for 15 min, and then the supernatant was collected. After reaction with Ellman's reagent (DTNB), GSH reductase enzyme, and NADPH, the absorbance was measured at 412 nm using a microplate reader (Tecan Infinite Pro, Switzerland).

### Statistical Analysis

Data were expressed as mean ± SEM and analyzed using GraphPad Prism software version 8.0. Comparisons between multiple groups were performed using one-way multivariate ANOVA (Tukey's *post-hoc* test). The difference was considered statistically significant when the *p*-value was ≤ 0.05.

## Results

### DOX-Induced Cardiac Dysfunction and Hypotrophy

**Figure 1:**
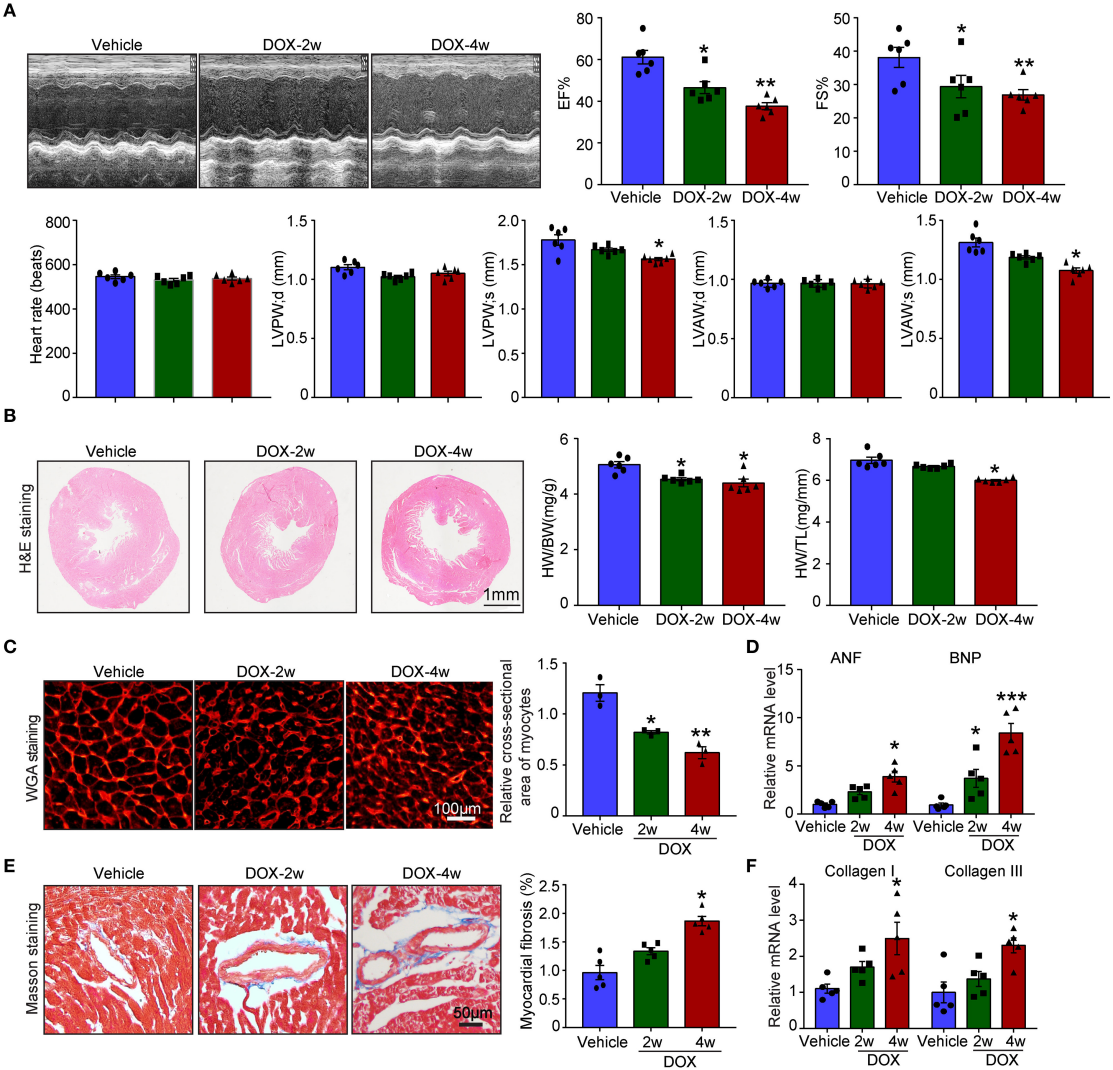
Doxorubicin (DOX) dose-dependently induced cardiac dysfunction and cardiac fibrosis. Mice were intravenously treated with DOX (5 mg/kg) or its solvent once per week for 2 or 4 weeks. **(A)** Representative M-model echocardiography and the measurement of left ventricular EF% and FS%. The heart rate, left ventricular posterior wall (LVPW), and left ventricular anterior wall (LVAW) at diastole and systole were shown below (*n* = 6). **(B)** Representative image of hematoxylin and eosin (H and E) staining and the HW/BW and HW/TL ratios. Scale bar 1 mm. **(C)** Cardiac myocyte size was evaluated by TRITC-labeled wheat germ agglutinin (WGA) staining, and the cross-sectional area was quantified in the right panel (100 cells counted per heart, *n* = 3). **(D)** ANF and BNP mRNA expressions were determined by qRT-PCR in the heart (*n* = 5). **(E)** Cardiac fibrosis was detected by Masson's trichrome staining, and the relative fibrotic area was quantified (*n* = 5, right). Scale bar 50 μm. **(F)** The collagen I and collagen III mRNA levels in the heart (*n* = 5). Data are presented as mean ± SEM, and n represents the number of animals per group. *p < 0.05, **p < 0.01, ***p < 0.001 vs. vehicle.

Numerous studies established the mice model of DOX-induced cardiotoxicity by intraperitoneal injection of DOX. However, intraperitoneal administration of DOX resulted in several side effects that influenced the accuracy of experimental conclusions. Intraperitoneal injection of DOX in mice severely reduced the food and water intake, induced peritoneal damage, and fibrosis which subsequently led to malaise, weight loss, and finally non-cardiac death. To avoid these inadequacies, intravenous injection of low dose DOX (5 mg/kg, once a week) for 4 weeks was used to induce cardiotoxicity. We found DOX time-dependently reduced cardiac function reflected by reduced LVEF, LVFS, LVPW, and LVAW during systole compared with vehicle-treated mice (Figure 1A). Furthermore, DOX dramatically reduced heart size, heart weight (HW) or body weight (BW), HW/tibia length (TL) ratio, and myocyte cross-sectional area (Figures 1B,C), whereas increased the mRNA expression of ANF and BNP (Figure 1D). Masson's trichrome staining showed that DOX treatment for 2- and 4-weeks time-dependently increased fibrotic area (Figure 1E); correspondingly, the mRNA level of fibrotic marker (collagen I and collagen III) in DOX-treated heart was also increased compared with that in the vehicle-treated heart (Figure 1F).

### Tail Intravenous Injection of DOX-Induced Myocardial Oxidative Stress and Apoptosis With Few Systemic Side Effects

**Figure 2:**
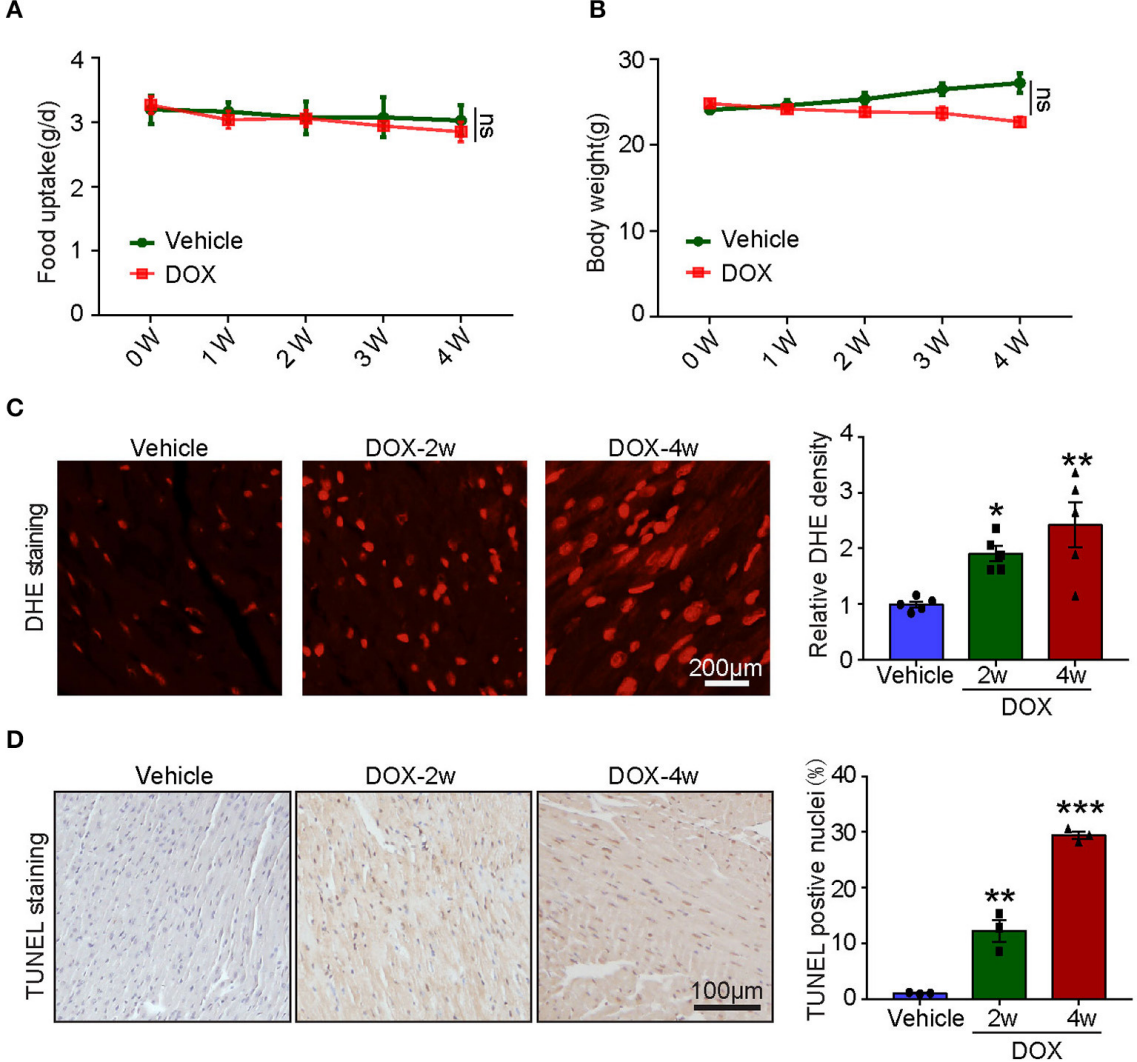
Doxorubicin dose-dependently induced cardiac oxidative stress and apoptosis. Mice were intravenously treated with DOX (5 mg/kg) or its solvent once per week for 2 or 4 weeks. **(A,B)** The changes of food uptake and body weight after DOX treatment (*n* = 4). **(C)** Reactive oxygen species (ROS) generation was measured by dihydroethidium (DHE) staining, and the quantification of fluorescence intensity was shown right (*n* = 5). Scale bar 200 μm. **(D)** Representative TUNEL staining in the heart tissues and the TUNEL-positive nuclei were quantified (*n* = 3, right). Scale bar 100 μm. *p < 0.05, **p < 0.01, ***p < 0.001 vs. vehicle.

To assess the systemic side effects of DOX, the food intake and body weight of the vehicle- and DOX-treated mice were recorded. We found that intravenous administration of DOX for 1–4 weeks had little effect on food intake and body weight (Figures 2A,B). Next, we examined ROS generation in the heart and found DOX treatment for 2- and 4-weeks time-dependently increased ROS generation reflected by the enhanced fluorescence intensity in the heart (Figure 2C). Meanwhile, TUNEL staining showed DOX markedly induced myocardial apoptosis as reflected by increased TUNEL-positive nuclei in the hearts (Figure 2D).

### Rut Inhibited DOX-Induced Cardiac Dysfunction and Fibrosis in Mice

**Figure 3:**
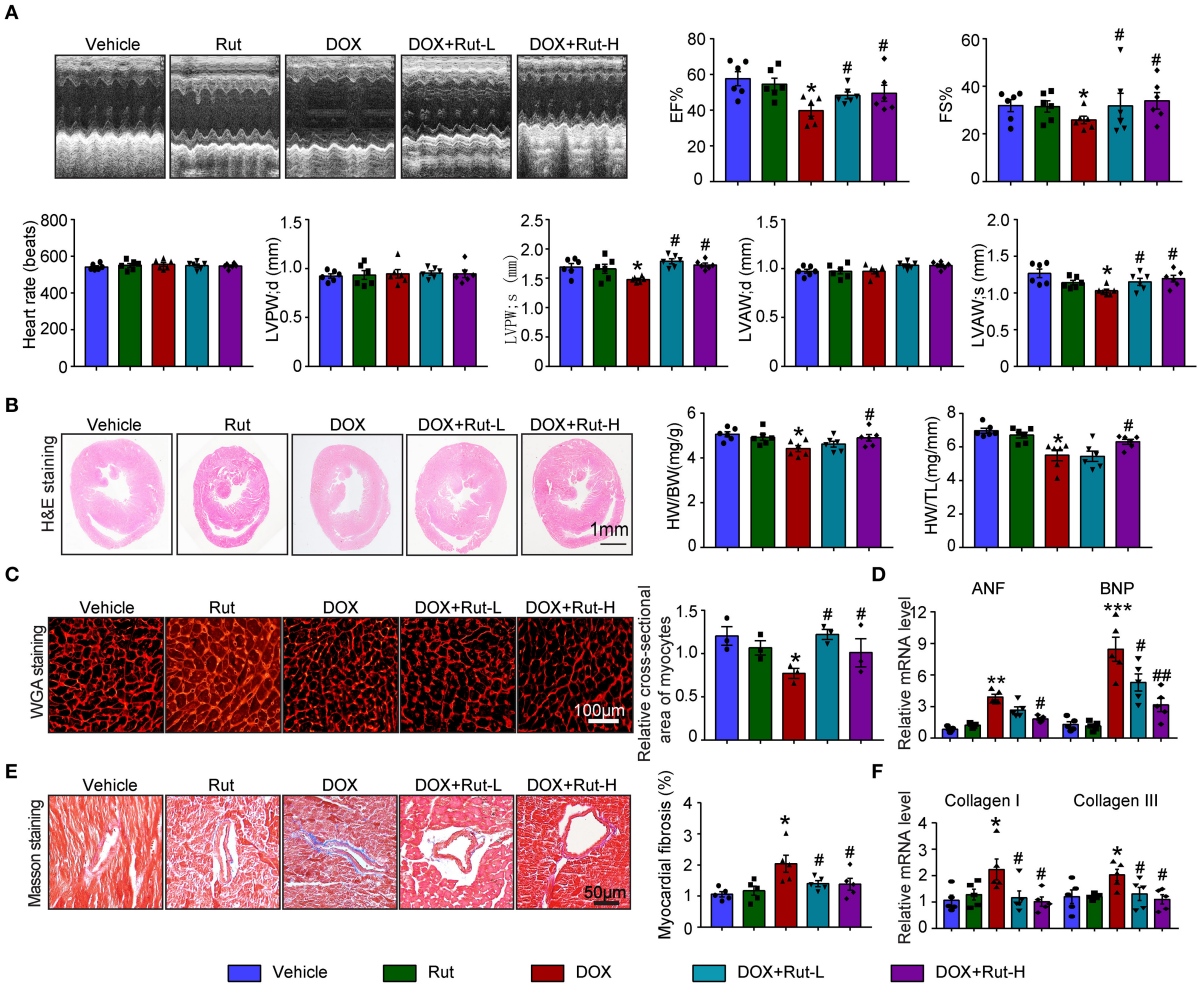
Rutaecarpine mitigated DOX-induced cardiac dysfunction and fibrosis. Mice were treated with DOX and then on the same day received intragastric administration of 20 or 40 mg/kg Rut every day for 4 weeks. **(A)** Representative M-model echocardiography and the measurement of left ventricular EF% and FS%. The heart rate, LVPW, and LVAW at diastole and systole were shown below (*n* = 6). **(B)** Representative image of HandE staining and the HW/BW and HW/TL ratios (right, *n* = 6). Scale bar 1 mm. **(C)** Cardiac myocyte size was evaluated by TRITC-labeled WGA staining, and the cross-sectional area was quantified in the right panel (100 cells counted per heart, *n* = 3). **(D)** ANF and BNP mRNA expressions were determined by real-time quantitative PCR (qRT-PCR) in the heart (*n* = 5). **(E)** Cardiac fibrosis was detected by Masson's trichrome staining, and the relative fibrotic area was quantified (*n* = 5, right). Scale bar 50 μm. **(F)** The collagen I and collagen III mRNA levels in the heart (*n* = 5). Data are presented as mean ± SEM, and n represents the number of animals per group. *p < 0.05, **p < 0.01, ***p < 0.001 vs. vehicle; ^#^*p* < 0.05, ^##^*p* < 0.01 vs. DOX group.

Since intragastric administration of Rut (low dose of 20 mg/kg and high dose of 40 mg/kg) exerted beneficial effects in cardiovascular diseases (13), we selected these doses of Rut to investigate whether Rut protected against DOX-induced cardiotoxicity. Echocardiography showed DOX-treated mice developed heart failure reflected by reduced LVEF, LVFS, LVPW, and LVAW during systole compared with vehicle-treated mice, and these detrimental effects were dramatically mitigated by Rut administration (Figure 3A). Furthermore, Rut inhibited DOX-induced cardiac hypotrophy as reflected by the increased ratios of heart weight/body weight (HW/BW) and heart weight/tibia length (HW/TL), myocyte cross-sectional area, and decreased mRNA level of ANF and BNP (Figures 2B–D). Results obtained from Masson's trichrome staining and qPCR analysis revealed that the cardiac fibrosis caused by DOX was also inhibited after low and high doses of Rut administration, indicating attenuated cardiac remodeling (Figures 3E,F). However, there existed no significant changes in heart function, cardiomyocytes size, and fibrosis between the vehicle and Rut group (Figures 3A–F).

### Rut Activated AKT/Nrf-2 Pathway and Inhibited DOX-Induced Oxidative Damage in the Heart

**Figure 4:**
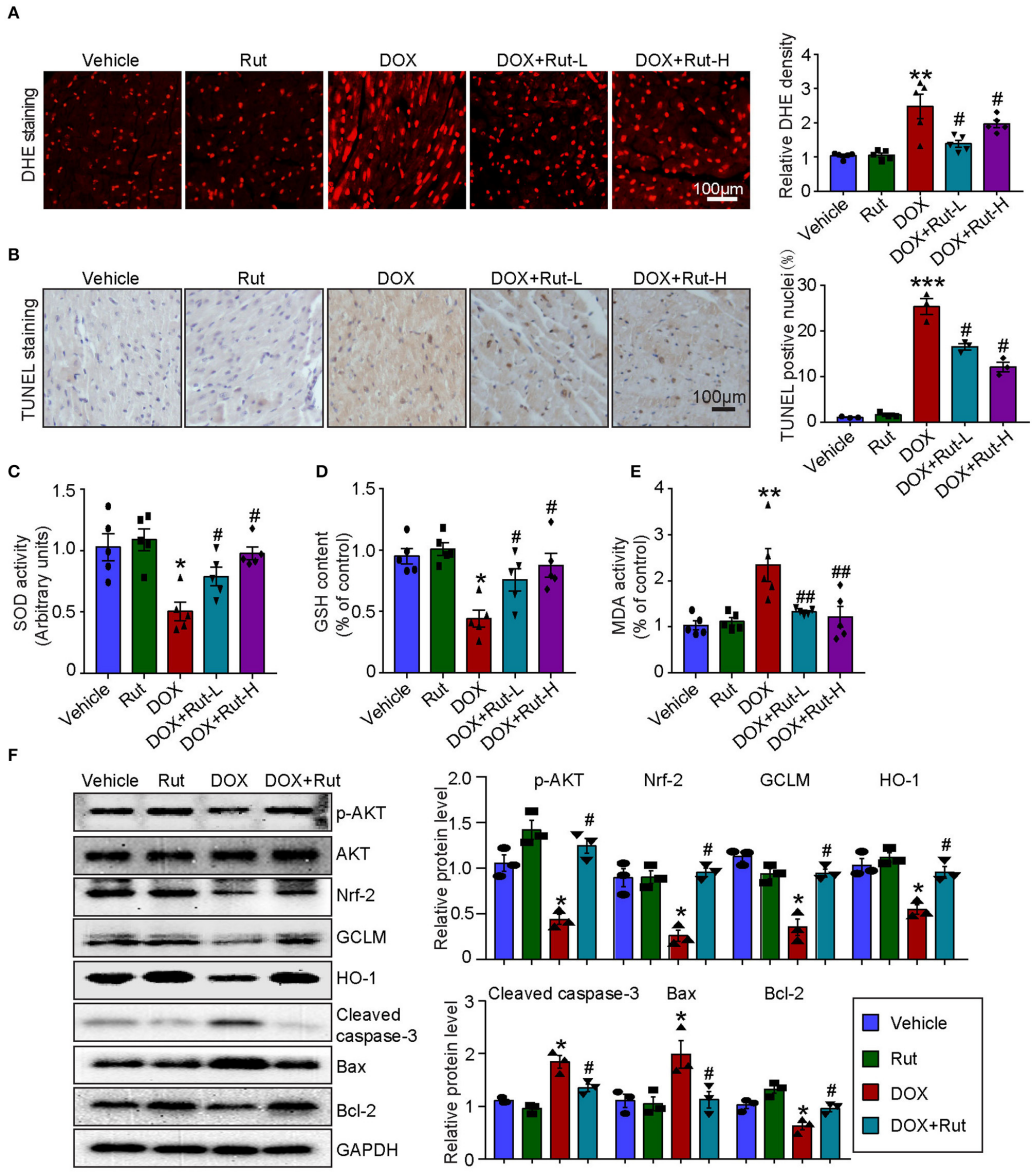
Rutaecarpine alleviated DOX-induced oxidative stress and apoptosis. Mice were treated with DOX and then on the same day received 20 or 40 mg/kg Rut (intragastric administration) every day for 4 weeks. **(A)** ROS generation in the heart was detected by DHE staining, and the quantification of fluorescence intensity was shown right (*n* = 5). Scale bar 100 μm. **(B)** Representative TUNEL staining in the heart tissues and the TUNEL-positive nuclei were quantified (*n* = 3, right). Scale bar 100 μm. **(C–E)** Relative superoxide dismutase (SOD) activity, glutathione (GSH) content, and enhancement of malondialdehyde (MDA) activity in the heart; data in other groups were normalized to the vehicle group (*n* = 5). **(F)** Western blot analysis of p-AKT, Nrf-2, GCLM, HO-1, cleaved caspase-3, Bax, and Bcl-2 in the heart and the relevant quantification was shown right (right, *n* = 3). *p < 0.05, **p < 0.01, ***p < 0.001 vs. vehicle; ^#^*p* < 0.05, ^##^*p* < 0.01 vs. DOX group.

PI3K/AKT/Nrf-2-mediated oxidative stress was the most important mechanism of DOX-induced cardiotoxicity (10); thus, in this study, we examined ROS generation, oxidant enzymes content and AKT/Nrf-2 protein expressions in DOX-treated hearts after Rut administration. DHE and TUNEL staining showed that Rut attenuated DOX-induced ROS generation and cardiomyocytes apoptosis in the heart (Figures 4A,B). Meanwhile, paralleled experiments demonstrated that DOX significantly reduced SOD activity and GSH content, but increased MDA activity in the heart, and all these effects were partially inhibited by Rut (Figures 4C–E).

To investigate the molecular mechanism of the antioxidant effects of Rut, we examined the protein levels of p-AKT, Nrf-2, HO-1, and GCLM in DOX-treated hearts after Rut administration and found that DOX reduced the protein level of p-AKT, Nrf-2, HO-1, and GCLM compared with vehicle-treated mice (Figure 4F), but the reduction in these protein expressions was reversed by Rut administration for 4 weeks (Figure 4F). In addition, the proteins involved in apoptosis were examined. Consistent with the results of TUNEL staining shown in Figure 4B, we found DOX enhanced the pro-apoptotic protein cleaved caspase-3 and Bax expressions, but reduced the antiapoptotic protein Bcl-2 expression (Figure 4F). Interestingly, these changes were all partly inhibited by Rut, suggesting the antiapoptotic effect of Rut (Figure 4F).

### AKTi Partially Reversed the Beneficial Effect of Rut on DOX-Induced Oxidative Stress and Apoptosis in the Heart

**Figure 5:**
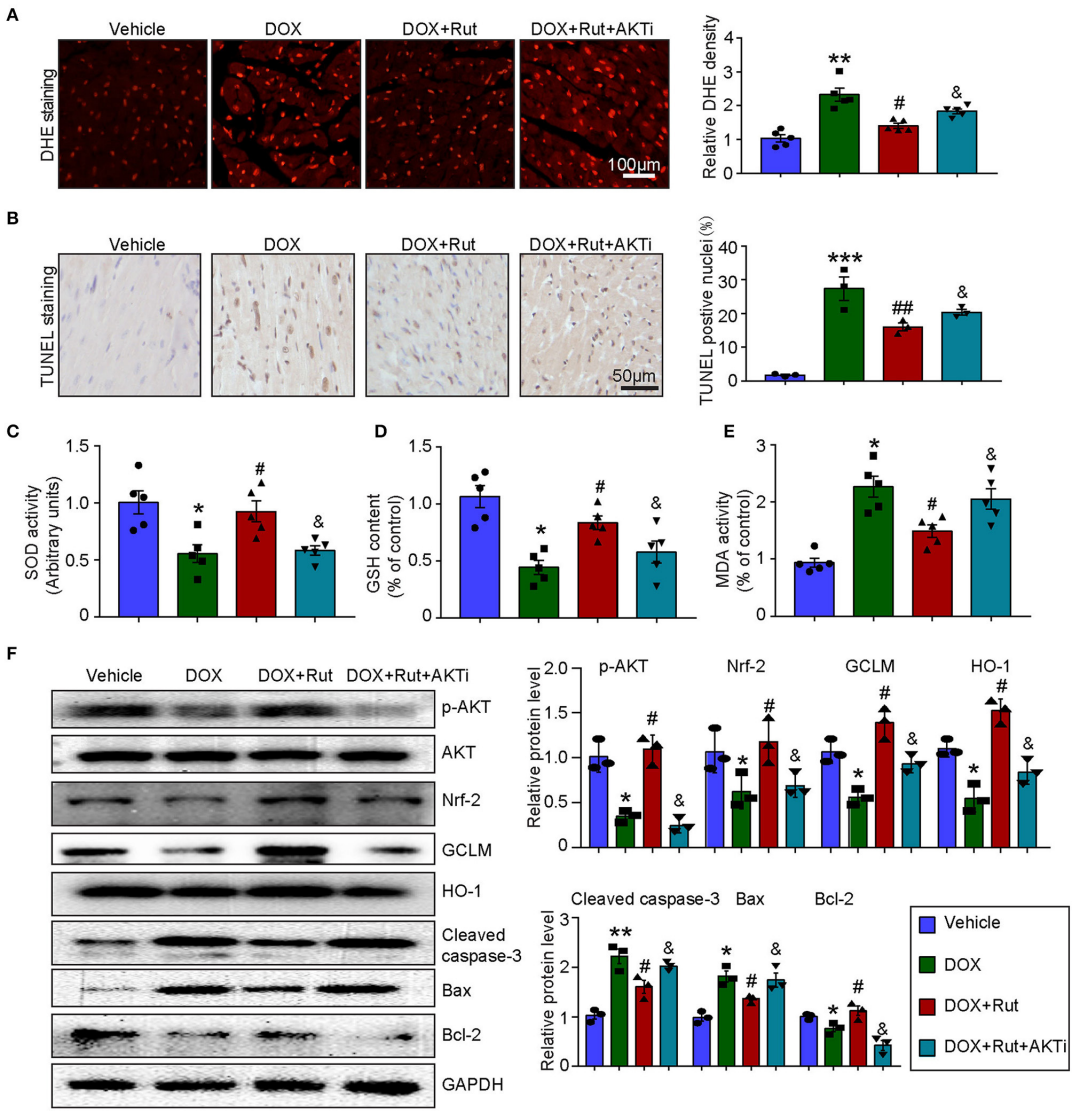
AKT inhibitor (AKTi) abolished the antioxidant and antiapoptotic effect of rutaecarpine in DOX-treated hearts. Mice were intraperitoneally injected with AKTi (20 mg/kg) every other day for 4 weeks before DOX and Rut administration. **(A)** ROS generation in the heart was detected by DHE staining, and the quantification of fluorescence intensity was shown right (*n* = 5). Scale bar 100 μm. **(B)** Representative TUNEL staining in the heart tissues and the TUNEL-positive nuclei were quantified (*n* = 3, right). Scale bar 50 μm. **(C–E)** Relative SOD activity, GSH content, and MDA activity in the heart; data in other groups were normalized to the vehicle group (*n* = 5). **(F)** Western blot analysis of p-AKT, Nrf-2, GCLM, HO-1, cleaved caspase-3, Bax, and Bcl-2 in the heart, and the relevant quantification was shown right (*n* = 3). *p < 0.05, **p < 0.01, ***p < 0.001 vs. vehicle; ^#^*p* < 0.05, ^##^*p* < 0.01 vs. DOX group; ^&^*p* < 0.05 vs. DOX plus Rut group.

To further confirm that the antioxidant effects of Rut were due to its ability to activate AKT/Nrf-2, the DOX combined with Rut-treated mice was subjected to AKTi. We found that the reduced ROS generation and apoptosis caused by Rut in DOX-treated heart were partially abolished by AKTi (Figures 5A,B). In concordance with these results, blocking AKT by its inhibitor counteracted the antioxidant effects of Rut, as demonstrated by the reduced SOD activity and GSH content, and increased MDA activity in the heart compared with DOX plus Rut group (Figures 5C–E). Accordingly, we examined the protein expressions which regulated oxidative stress and apoptosis in the heart and found that Rut enhanced the protein levels of p-AKT, Nrf-2, HO-1, GCLM, and Bcl-2, but inhibited cleaved caspase-3 and Bax expression compared with DOX-treated mice. Of note, all these changes in protein expressions were reversed after AKTi administration (Figure 5F), suggesting that the antioxidant and antiapoptotic effects of Rut were mediated by the AKT signal pathway.

### AKTi Partially Abolished the Protective Effect of Rut on DOX-Induced Heart Failure

**Figure 6:**
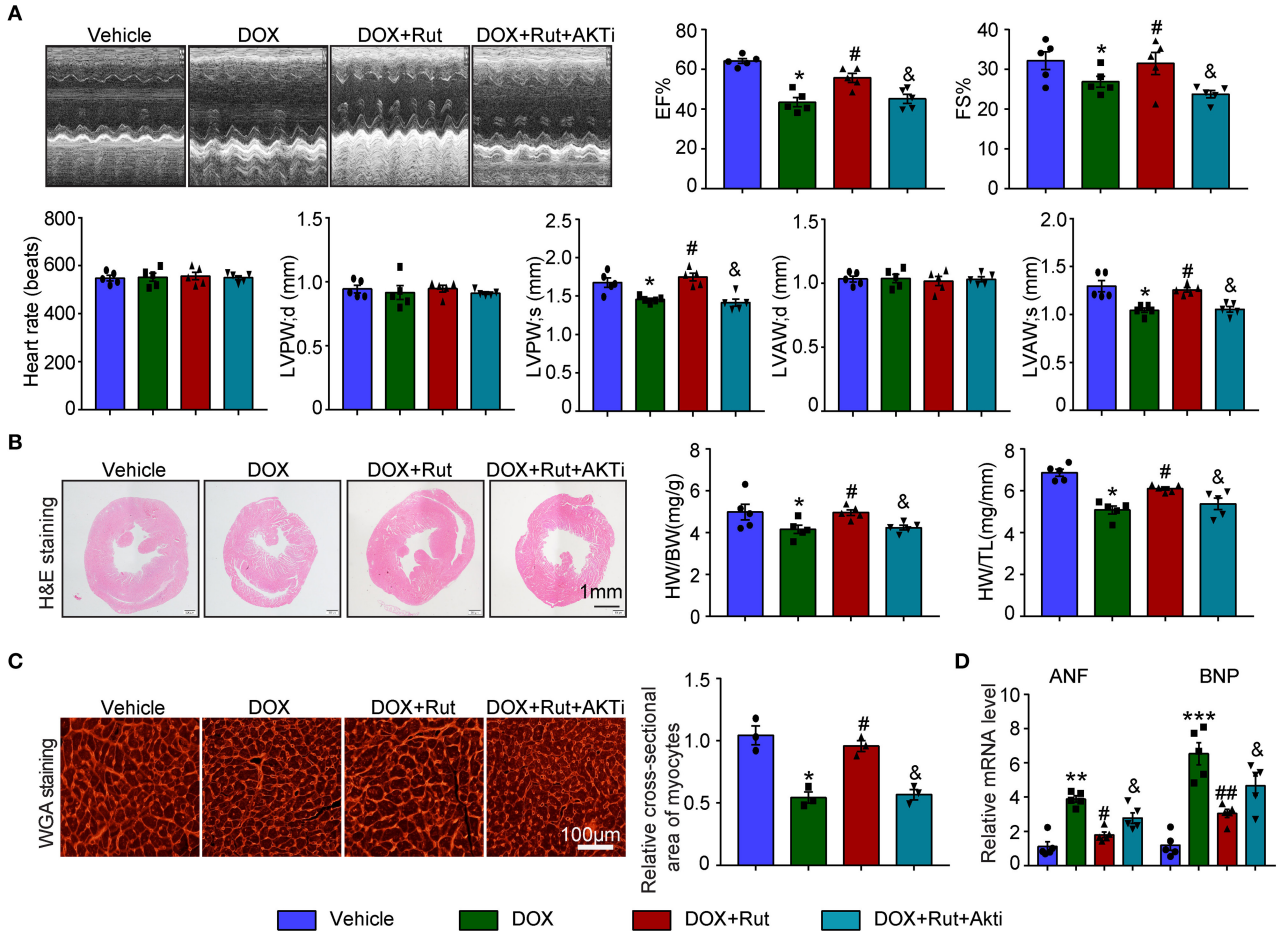
AKT inhibitor alleviated the cardio-protective effect of rutaecarpine on DOX-induced heart failure. Mice were intraperitoneally injected with AKTi (20 mg/kg) every other day for 4 weeks from the first day before DOX and Rut administration. **(A)** Representative M-model echocardiography of the left ventricular EF% and FS%. The heart rate, LVPW, and LVAW at diastole and systole were shown below (*n* = 5). **(B)** Representative image of HandE staining and the HW/BW and HW/TL ratios (right, *n* = 5). Scale bar 1 mm. **(C)** Cardiac myocyte size was evaluated by TRITC-labeled WGA staining, and the cross-sectional area was quantified in the right panel (100 cells counted per heart) (*n* = 3, right). Scale bar 100 μm. **(D)** ANF and BNP mRNA expressions were determined by qRT-PCR in the heart (*n* = 5). *p < 0.05, **p < 0.01, ***p < 0.001 vs. vehicle; ^#^*p* < 0.05, ^##^*p* < 0.01 vs. DOX group; ^&^*p* < 0.05 vs. DOX plus Rut group.

Next, the changes in cardiac function and structure were examined. We found that DOX-treated mice after Rut administration exhibited improved cardiac function, as reflected by increased LVEF, LVFS, LVPW, and LVAW during systole and also enhanced HW/BW and HW/TL ratios and myocyte size (Figures 6A–C), and these beneficial effects of Rut were partially inhibited by AKTi (Figures 6A–C). At the molecular level, we found that the biomarker of heart failure (ANF and BNP mRNA) enhanced in DOX-treated hearts was correspondingly reduced after Rut treatment, and AKTi partially reversed these effects (Figure 6D). Hence, these *in vivo* data suggested that Rut inhibited DOX-induced cardiotoxicity by activating AKT.

## Discussion

**Figure 7:**
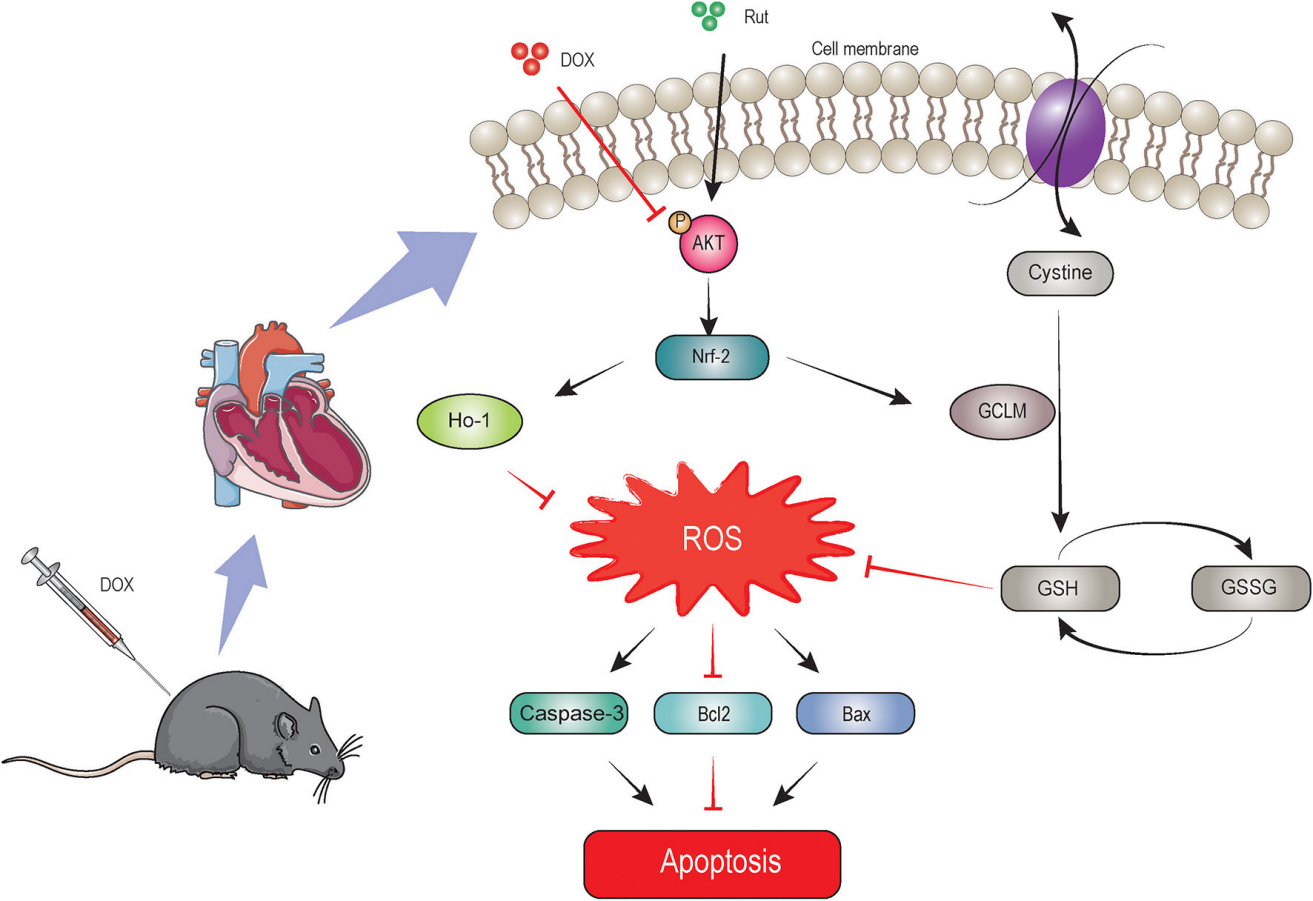
The proposed protective mechanism by rutaecarpine on DOX-induced heart failure. Rutaecarpine activated AKT/Nrf-2 signaling pathway, which in turn inhibited DOX-induced oxidative stress and apoptosis through regulating heme oxygenase-1 (HO-1) and GSH cysteine ligase modulatory subunit (GCLM) expression.

In this study, we identified Rut as a novel drug in treating DOX-induced cardiotoxicity, which was supported by inhibition of oxidative stress, myocardial apoptosis, fibrosis, and improvement of cardiac function. Rut enhanced HO-1 and GCLM expressions thereby leading to reduced ROS generation through activating the AKT/Nrf-2 signaling pathway, which was responsible for the beneficial effects of Rut on DOX-induced cardiotoxicity, and AKTi abolished these effects. These effects were summarized in Figure 7. These results highlighted that Rut may be a potential therapeutic drug for DOX-induced heart failure.

Doxorubicin is an effective chemotherapeutic drug in treating multiple cancer all over the world, but as we know, this action also comes at a price. Numerous patients who received DOX treatment developed heart failure, and this detrimental side effect even occurs many years after the cessation of DOX administration. Although there existed a cardioprotective drug that alleviated DOX-induced cardiotoxicity such as dexrazoxane, the curative effect was still not satisfactory. Thus, searching for novel drugs to mitigate the cytotoxicity of DOX and revealing the underlying mechanism is an important task. Recent studies suggested that Rut has antitumor potential by inhibiting proliferation and inducing apoptosis in multiple tumor cells, including human ovarian cancer cells (22), breast cancer cells (23), and prostate cancer cells (24). On the other side, Rut had cardioprotective roles in several cardiovascular diseases including hypertension (12), atherosclerosis (25), cardiac hypertrophy (13), and ischemia-reperfusion injury (14). However, whether Rut protected against DOX-induced cardiotoxicity remains obscure. In this study, our *in vivo* data showed that Rut inhibited DOX-induced cardiac dysfunction, oxidative stress, myocardial apoptosis, and loss of heart weight. These results indicated that Rut may be a potential drug to treat cancer and meanwhile prevent DOX-induced cardiotoxicity.

AKT signaling pathway plays an important role in regulating oxidative stress and apoptosis, and activation of AKT exerts protective effects on DOX-induced cardiotoxicity (10). Oxidative stress was able to trigger cardiomyocyte apoptosis and was one of the most important mechanisms of DOX-induced cardiotoxicity (3). Recent studies reported DOX reduced antioxidant substances such as GSH and SOD and induced apoptosis which was accompanied by increased cleaved caspase-3, Bax, and decreased Bcl-2 expression (10). GSH functions at catalyzing the reduction of peroxides, and SOD promoted the transition of O^2−^ to hydrogen peroxide (26, 27). Consistent with these reports, our results showed that the level of GSH and SOD was reduced in DOX-treated hearts, but the level of MDA (the end product of lipid hydroperoxide) was increased. Additionally, the protein which regulated apoptosis manifested similar changes upon DOX treatment, as demonstrated by upregulation of cleaved caspase-3 and Bax and downregulation of Bcl-2 protein expressions in DOX-treated hearts. Importantly, Rut administration for 4 weeks dramatically alleviated all these effects. Of note, AKT/Nrf-2/GCLM signaling pathway inactivated by DOX was reactivated by Rut. To get deeper knowledge on the mechanism of Rut protected against DOX-induced cardiotoxicity, AKTi was then used to block AKT signaling pathway. Our results showed that AKTi inhibited AKT/Nrf-2/GCLM signaling pathway activated by Rut and further abolished the beneficial effects of Rut in DOX-induced cardiotoxicity. These results suggested that Rut may inhibit DOX-induced cardiotoxicity through AKT/Nrf-2/GCLM-mediated oxidative stress and apoptosis.

However, some limitations still existed in this study. These *in vivo* experiments only used mice to investigate the effects of Rut, and more animal models of DOX-induced cardiotoxicity and clinical experiments should be established to further confirm the protective role of Rut. In addition, although existing evidence indicated that Rut activated AKT signaling pathway through calmodulin-dependent protein kinase-II or IRS-1/PI3K in HepG2 cell or liver tissues, respectively (15, 18), whether these mechanisms were similar in the heart needs to be studied in future.

In summary, we discovered a novel drug that protected against DOX-induced cardiotoxicity. The molecular mechanism may involve the activation of the AKT/Nrf-2/GCLM signaling pathway, which alleviates DOX-induced oxidative stress and apoptosis to improve cardiac dysfunction. These findings may provide a novel therapeutic option in the treatment of DOX-induced cardiotoxicity. These findings indicated that Rut was a cardio-protective agent in treating DOX-induced cardiac toxicity.

## Data Availability Statement

The raw data supporting the conclusions of this article will be made available by the authors without undue reservation.

## Ethics Statement

The animal study was reviewed and approved by the Animal Care and Use Committee of the Dalian Medical University.

## Author Contributions

Z-QL, Z-LS, J-TL, C-LL, and H-LB participated in most of the experiments. Z-QL, H-LB, and Z-LS performed the relevant experiment of molecular biology. X-LY, Z-QL, and Z-LS performed animal studies. XX, YZ, and Y-NJ conceived and supervised the project. XX and X-LY wrote the manuscript. All authors contributed to the article and approved the submitted version.

## Conflict of Interest

The authors declare that the research was conducted in the absence of any commercial or financial relationships that could be construed as a potential conflict of interest.

## Publisher's Note

All claims expressed in this article are solely those of the authors and do not necessarily represent those of their affiliated organizations, or those of the publisher, the editors and the reviewers. Any product that may be evaluated in this article, or claim that may be made by its manufacturer, is not guaranteed or endorsed by the publisher.
